# Penny-wise and pound-foolish

**DOI:** 10.1007/s00167-018-4852-3

**Published:** 2018-02-09

**Authors:** Asbjørn Årøen, Brian M. Devitt

**Affiliations:** 1 Department of Orthopedic surgery, Institute of clinical Medicine, Akershus University Hospital, Campus Ahus University of Oslo, Oslo, Norway; 2 Oslo Sports Trauma center, NIH, Oslo, Norway; 3 OrthoSport Victoria, Epworth Richmond, 89 Bridge road, Richmond, VIC 3121 Australia


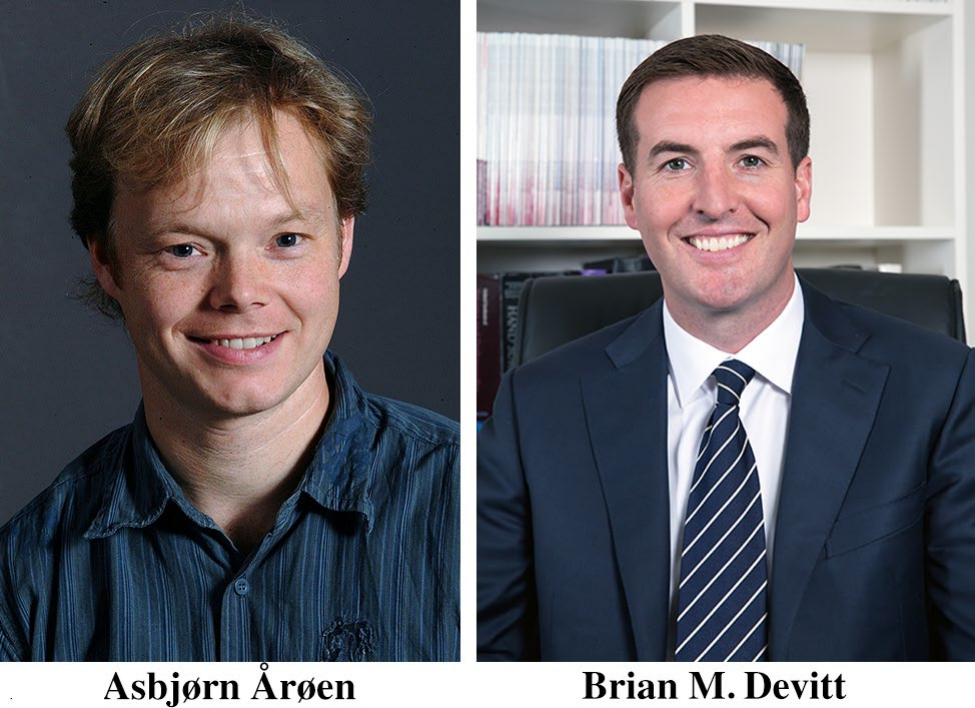
This issue of the KSSTA journal has a special section on concurrent anterior cruciate ligament (ACL) and cartilage injuries. These two conditions are very common problems and the treatment of which represents a significant financial burden not only on healthcare systems internationally but also on society in general. As resources in healthcare are not unlimited, we, as orthopedic surgeons, must be conscious of the cost of surgery and rehabilitation we provide for our patients. But, it is not simply a measure of the monetary cost that it is important, it is the cost to benefit ratio that gives us a better impression of the overall value of the treatment we provide. As the British saying goes do we run the risk of being ‘penny-wise and pound-foolish’ by trying to cut corners on the initial cost of treatment only to pay dearly in terms of lost productivity and chronic disability in the long-run?

In the context of cartilage surgery, given that the patient population is typically young, the cost of treatment must be considered against the societal saving of returning these patients to gainful employment. It has previously been shown that patients with focal cartilage lesions suffer with significant symptoms of pain and functional impairment and their quality of life is affected to the same extent as patients scheduled for a total knee arthroplasty (TKA) [6]. Therefore, considering the price of a TKA exceeds that of most cartilage restoration procedures, should we consider the investment in a biological alternative as money well spent no matter the cost [10]? On the other hand, some might argue that whereas TKA is typically a very reliable procedure and delivers value for money, the same is not always true of cartilage restoration procedures in the knee.

Milton Friedman, a nobel prize winning economist, in a famous interview on the ways to spend money outlined an important distinction about one’s attitude to spending based on who the money belongs to, which is quite relevant in this discussion: *“You can spend your own money on yourself. When you do that, why then you really watch out what you’re doing, and you try to get the most for your money… And if I spend somebody else’s money on somebody else, I’m not concerned about how much it is, and I’m not concerned about what I get. And that’s government*.” With this sentiment in mind, let us consider two scenarios of how we would spend our own money if we had to pay to treat the two following knee conditions: (1) An isolated 2 cm × 2 cm cartilage lesion of the medial femoral condyle in a 28-year-old male patient with normal alignment: and (2) A combined ACL rupture with the same cartilage lesion in the same patient.

Scenario 1—An isolated chondral lesion of the medial femoral condyle.

First, let us examine the treatment options and see which one is the best regardless of the expense. In this instance, we are spoilt for choice as there are a myriad of treatment options available. As in most circumstances where there are so many options, a superior option usually does not exist and this is certainly true in this setting. In a systematic review of high quality, randomized control trials comparing cartilage repair techniques, no single most effective treatment could be determined [5]. Nonetheless, regardless of the type of cartilage regeneration technique used, an improvement in the measured clinical outcome was observed compared to pre-surgical baseline levels. So, all treatments seem to be better than nothing, but how sustainable are they?

In considering the effectiveness of cartilage treatment, it is important not to focus solely on the short-term results of treatment but to examine the longer-term follow-up and especially the failure rates associated with the various treatment methods. Vanlauwe et al. reported a significant improvement in KOOS for characterized chondrocyte implantation (CCI) versus microfracture at 3 years of follow-up, but no difference could be found at 5 years; the failure rates were also comparable at 13.7 and 16.4%, respectively [20]. Knutsen et al. were also unable to detect a difference in clinical outcome or failure rate after 15 years comparing autologous chondrocyte implantation (ACI) and microfracture [7]. Reporting on the 9.8-year results comparing OAT to microfracture, Ulstein et al. found no significant difference in KOOS scores between the groups [18]. So, if the outcomes of treatments are largely equivalent at long-term follow-up perhaps cost analysis could sway the argument in our treatment choice?

In this issue, Aae et al. performed a cost analysis comparing microfracture and ACI. The results of four high quality studies, with a follow-up of 5 years, revealed that microfracture was more cost-effective when comparing all clinical scores. Both groups achieved substantially better clinical scores at 5 years compared to baseline but the reoperation rate was 13.5% and 12.1% for microfracture and ACI, respectively [1]. Schrock et al. in a similar cost analysis of ACI, osteochondral autograft transplant (OAT) and microfracture determined that microfracture was the most cost effective but the next-generation ACI using a biological or engineered scaffold had a statistically significant functional improvement [14]. Interestingly, the cost-per-point change in functional outcome score was > 50% greater for OATs compared to microfracture, but substantially more for ACI at > 150%. Finally, Mistry et al. in a comprehensive systematic review and economic evaluation of ACI concluded that ACI was cost-effective, taking into account short-term improvements in symptoms and the reduced need for further repairs and, in the long term, knee replacements [9]. The conclusion was based on the findings of four high quality randomized controlled trials with microfracture as the comparative treatment in all cases. The economic modelling used measured cost per quality adjusted life years (QALY) as opposed to cost-per-point change in functional outcome which had been used in the former two studies.

One of the hidden expenses that must also be considered is the cost of rehabilitation. In the setting of microfracture, the recommended rehabilitation regime is quite detailed and rigorous; Dr Richard Steadman, who devised this technique, recommended continuous passive motion postoperatively in his rehabilitation protocol for 6- to 8-hours every 24-hours [15, 17]. This rehabilitation regimen is certain to add to the cost of the procedure, but the developing surgeons claim that failure to adhere to the appropriate rehabilitation regimen is the reason for substandard results [16]. Interestingly, the study by Aae et al. considered the cost of rehabilitation in their analysis, which was not accounted for in the study by Mistry et al [1, 9]. In the latter’s economic assessment, the authors assumed that assessment costs and rehabilitation costs were identical between the treatment groups in the randomized controlled trials, so they were not included in the comparison.

Another factor to consider in the cost of cartilage restoration surgery is the length of the inpatient stay in hospital. This is perhaps a further reason for the discrepancy in two cost analyses between the studies by Aae et al. and Mistry et al [1, 9]. Inpatient stay in hospitals are expensive, and there is no doubt that if ACI can be performed as day-surgery the cost will be significantly less, as shown in the report by Mistry et al. However, day surgery is not typically the norm for standard chondrocyte implantation and for this reason the manuscript by Aae et al. has reached different economic conclusions. It also demonstrates that small changes in the technique could result in major changes in the costs involved and future development will surely reduce the expense. However, at present we must continue to use standard, well-documented techniques to determine the costs when comparing techniques until these future techniques become a reality.

Scenario 2—A combined ACL rupture and an isolated 2 cm × 2 cm cartilage lesion of the medial femoral condyle.

At the moment, we are still far from understanding the exact cause of cartilage injuries of the knee except in the case of major ligamentous injury such as ACL rupture. Whether the cartilage insult is sustained at the time of the ligamentous injury or subsequently as a result of knee instability the risk of cartilage injury to an ACL deficient knee is significantly increased [4]. It has also been shown that inferior patient reported outcomes have been recorded when chondral pathology is present at the time of reconstruction surgery [11, 12]. Interestingly, it has also been reported that the treatment of cartilage lesions in the setting of ACL reconstruction with microfracture results in inferior 5 year functional outcomes compared to simply stabilization of the lesions [13]. It would appear that in the setting of concomitant ACL and cartilage injury, the treatment of instability with ACL reconstruction is the critical factor and not necessarily addressing the chondral lesion. Ulstein et al. have shown that ACL reconstruction performed in patients with an isolated concomitant full-thickness cartilage lesion restored patient-reported knee function to the same level as ACL reconstruction performed in patients without concomitant cartilage lesions, 5–9 years after surgery [19]. As such, perhaps ‘*less is more*’ in this group and a more minimalist approach to treating the chondral lesion should be adopted.

Knowledge is of no value unless you put it into practice

- Anton Chekov

As surgeons, it is incumbent on us to be aware of the cost of treatment. This attitude will not only lead to better value for money but will encourage the development of alternative solutions to current problems. We need to be mindful of the appeal and enticement of novel treatments which offer the elusive solution to such a longstanding problem of chondral lesions of the knee. Even more so, seeing as the exposure to new products through our meetings, journals, and the internet, has never been higher. In fact, it is probably wise to maintain a healthy degree of skepticism until evidence is available to support its effectiveness of a particular treatment.

That is not to say that we should take an entirely miserly stance to new technology which could stymie advancement. However, expense ought to be justified prior to widespread implementation or recommendation of new products. In regions of plentiful resources, such as northern Europe or North American, it is likely there will be a greater number of early adopters of new techniques and as a global orthopaedic community we rely greatly on these groups to critically appraise the efficacy of these technique in a rigorous scientific manner. Most society will find room for this technology if it is clearly evidence based and long-term results are superior.

In summary, it has been shown through several official cost analyses that musculoskeletal injuries are one of the most common causes of absence from work in the working population [2, 3, 8]. As such, orthopaedic journals, which are the major contributors of scientific evidence in this field, have an important social and moral responsibility in promulgating this information. The KSSTA journal, as one of the key players in this area for almost three decades, will surely continue its central role in publishing high-level scientific studies to provide information on not only the best treatment but also the most cost effective ones. The topics highlighted in the current issue involving treatment of cartilage injury of the knee and ACL injuries, will in future be reviewed more closely based a costs to benefit analysis by the healthcare providers. If we, as experts in the field, not are able to manage the costs involved those holding the purse-strings will; clinical decisions made by surgeons will be overruled by economic decisions made by pencil-pushers. Currently, rigorous medical evidence stands as the best justification for the selection of a particular treatment and long may this continue. To achieve this costs analyses must be performed in conjunction with any evolving treatment strategies.
